# Sustainable Bio-Based Adsorbents for Simultaneous and Efficient Removal of Hazardous Dyes from Aqueous Solutions

**DOI:** 10.3390/toxics12040266

**Published:** 2024-04-01

**Authors:** Dhwani Vara, Stuti Jha, Shweta Bisht, Syed Shahabuddin, Rama Gaur, Suhas, Inderjeet Tyagi

**Affiliations:** 1 Department of Chemistry, School of Energy Technology, Pandit Deendayal Energy University, Knowledge Corridor, Raisan, Gandhinagar 382426, Gujarat, India; dhwani.vara@gmail.com (D.V.); stutijha97@gmail.com (S.J.); syed.shahabuddin@sot.pdpu.ac.in (S.S.); 2 Division of Research and Innovation, School of Applied and Life Sciences, Uttaranchal University, Dehradun 248007, Uttarakhand, India; swtbisht23@gmail.com; 3 Department of Chemistry, Gurukul Kangri (Deemed to be University), Hardwar 249404, Uttrakhand, India; suhasnatyan@yahoo.com; 4 Centre for DNA Taxonomy, Molecular Systematics Division, Zoological Survey of India, M-Block, New Alipore, Kolkata 700053, West Bengal, India

**Keywords:** adsorption, bio-based adsorbent, cationic dyes, wastewater remediation

## Abstract

Dyes provide a notable environmental issue as a result of their intrinsic poisonous and carcinogenic characteristics. An estimated 60,000 metric tons of dyes has been discharged into the environment, leading to a substantial increase in water pollution. The mitigation of these dyes is a substantial and intricate challenge. The primary objective of this research is to conduct a comprehensive analysis of the adsorption of cationic dyes containing positively charged groups such as sulphonates, amines, and triphenylmethanes. The adsorption study was carried out using four different low-cost adsorbents derived from biowaste, specifically Groundnut Shell (GS), Mosambi Peel (MP), Mango Bark (MBARK), and Mango Leaves (ML). The adsorbent materials were characterized using FTIR, UV–Vis spectroscopy, scanning electron microscopy (SEM), point-of-zero charge (PZC), and BET techniques. The adsorption capacity was found to be between 1.5 and 2.2 mg/gm for Groundnut Shell, Mosambi Peel, Mango Bark, and Mango Leaves for individual dye removal (Crystal violet, Methylene blue, Rhodamine B, and Malachite green). It was observed that adsorbent derived from mango bark showed excellent adsorption (%) in a mono-component dye system and, thus, was explored for the simultaneous removal of a mixture of the same dyes. MBARK exhibited an excellent overall dye removal efficiency of 94.44% (Q_e_ = 2.7 mg/g) for the dye mixture in 60 min. From a detailed kinetic investigation, it was concluded that the adsorption followed the pseudo-second-order model (R^2^= 0.99963 to 1 for different dyes and adsorbents) hinting at chemisorption. The effect of the pH of the analyte solution and the dosage of adsorbent was also studied for simultaneous removal. The isothermal studies demonstrated that the Langmuir adsorption model (R^2^ = 0.99416) was the best-fitted model, suggesting monolayer adsorption. The adsorption process was predicted to be governed by ion exchange, electrostatic interaction, hydrogen bonding, pi–pi interaction, etc., based on charge, functional groups, and pH of dyes and adsorbent. Thus, this study highlights the application of low-cost biowaste as a potential adsorbent for the mitigation of toxic industrial dyes present in wastewater.

## 1. Introduction

The issue of environmental contamination arose as a consequence of the rapid processes of industrialization and urbanization. The rate at which the concentration of toxins is discharged from several industrial sectors, such as oil refineries, paper manufacturers, textiles, and sugar mills, is escalating at a concerning pace [1]. These pollutants mainly consist of hazardous chemicals like heavy metals, (Cd, Cr, Hg, Cu, Ni, Zn), cyanides, toxic dyes (Crystal violet (CV), Methylene blue (MB), Rhodamine B (RhB), Malachite green (MG, etc.), oil, and various organic molecules (phenol, hydrocarbons) [2]. Therefore, the management and treatment of wastewater is a multifaceted and demanding matter that necessitates meticulous attention in order to safeguard both human health and the environment. Various physical and chemical techniques are employed in the treatment of wastewater, including ion exchange, chemical precipitation, coagulation/flocculation, flotation, membrane filtration, and adsorption, as outlined by Darban et al. in their research publication [3]. Table 1 provides a comprehensive analysis of the advantages and disadvantages associated with the various methodologies employed in the study.

Adsorption is a technology commonly employed in wastewater treatment due to its cost-effectiveness, simplicity, high efficiency, and widespread application [9]. The literature contains reports regarding the utilization of adsorbents such as zeolites, activated carbon, biochar, nanocomposites, and others [10,11,12,13,14]. Nevertheless, the majority of these adsorbents are either costly or necessitate an intricate procedure for production and processing, prompting researchers to seek a more cost-effective alternative [15]. In recent decades, there has been significant interest in biowaste-based adsorbents due to their several advantages, including cost-effectiveness, absence of sludge production, and straightforward processing procedures. Furthermore, the utilization of biowaste as an adsorbent makes a significant contribution to the field of waste management by facilitating the recycling of environmental waste. The primary constituents of these biowastes include cellulose, lignin, and hemicellulose, among others. These components possess various functional groups such as phenols, methoxy, hydroxyl, and carboxyl groups. These functional groups play a crucial role in the adsorption process by engaging in interactions with the analyte, such as hydrogen bonding, electrostatic interaction, and pi–pi interaction [16,17]. Biowaste has the potential to undergo conversion into biochar, a substance characterized by its high carbon content and charcoal-like properties. The utilization of biochar as an adsorbent has demonstrated promising solutions in the field of dye mitigation, mostly attributed to its exceptional physiochemical properties [18].

Real wastewater is a complex mixture containing multiple pollutants, and very few studies showing the removal of pollutants from a multi-component system are available in the literature as summarized in Table S1 (Supplementary Materials). Moreover, the reported studies either involve, costly activation steps, high adsorbent dosage, low removal efficiency, or are time-consuming [19,20,21,22,23,24,25,26,27,28,29,30,31,32,33,34,35,36,37,38,39,40,41].

Keeping in view the same, in the present study, we applied a low-cost adsorbent from the rapid remediation of noxious dyes, namely, Crystal violet, Methylene blue, Rhodamine B, and Malachite green from a single and multi-component aqueous system in order to assess the removal efficiency of the developed adsorbent. The expected simultaneous presence of these dyes is between 10 and 50 mg/L in real wastewater. The functional groups present on the surface of dyes (-RNH_2_, -SO_3_H, -COOH, and aromatic groups) and adsorbents are expected to interact with each other through hydrogen bonding, ion exchange, complexation, electrostatic forces, etc., occurring at the water (dye solution)–solid (adsorbent) interface, resulting in adsorption. Further, the impact of effective parameters such as pH, adsorbent dosage, contact time, etc., on the adsorption efficiency of the developed adsorbent was also investigated.

## 2. Materials and Methods

### 2.1. Materials

In the present study, the biowastes, i.e., Groundnut Shells (GS), Mosambi Peels (MP), Mango Bark (MBARK), and Mango Leaves (ML), were collected from the local market of Gandhinagar and its surroundings. Other chemicals: Methylene blue (Merck, 99% pure, Mumbai, India), Crystal violet (Merck, 99% pure, Mumbai, India), Rhodamine B (SRL, 99% pure, Mumbai, India), Malachite Green (Finar, 99% pure, Ahmedabad, India), Acetone (Finar, AR grade, 99% purity, Ahmedabad, India), NaNO_3_ (Merck, ACS grade, 99% purity, Mumbai, India), HCl (Finar, AR grade, 37% purity, Ahmedabad, India), and NaOH (Merck, ACS grade, 97% purity, Mumbai, India) were used as obtained. Distilled water was used for the adsorption studies.

### 2.2. Characterization

The collected materials were washed with distilled water to remove the dirt and other impurities. All the biowastes were then shade-dried. Further, the dried biowastes were ground using a grinder (550 W, Boss company, Ahemdabad, India). The grinder was operated for 1 h to grind 1 kg of adsorbent. Therefore, the cost was estimated to be 3.83 INR (0.048 USD/kg). The ground biowaste was sieved using a sieve to obtain fine powder of uniform size (<75 µm). After the pre-treatment of the biowastes, they were examined for morphological analysis, functional group analysis, surface area, particle size, and surface charge analysis with the help of different techniques like Scanning Electron Microscopy (SEM), Fourier Transform Infrared Spectroscopy (FT-IR), Brunauer–Emmett–Teller (BET), Zeta Sizer, and point-of-zero charge (PZC), respectively. The concentration of analyte was monitored using Ultraviolet–visible spectroscopy (UV–Vis). For FE-SEM analysis, the samples were dispersed on an aluminum stub over conducting carbon tape. To make the samples conductive in nature, they were coated with gold layer using LEICA EM ACE200. For IR analysis, the samples were analyzed using FT-IR-Perkin Elmer Spectrum 2 equipment operating in the ATR mode, for a scan range of 400 cm^−1^ to 4000 cm^−1^. Brunauer–Emmett–Teller (BET) measurements were carried out using nitrogen adsorption and desorption isotherms with 80 °C degassing temperature for 30 min. Zeta potential was measured on Zeta-90Plus particle size analyzer (Brookhaven Instruments Corporation, New York, United States) using a suspension of different samples prepared in water. For the PZC analysis, the salt addition method was used [42]. The UV–Vis spectroscopy analysis was performed using the PerkinElmer UV/VIS LAMBDA 365+ model (Ahemdabad, Gujarat, India) in wavelength scan and absorbance mode. The values of highest wavelength measurements (λ_max_) in mono-component for CV, MB, RhB, and MG were 582, 663, 554, and 618 nm, respectively, and in multi-component systems the values of highest wavelength measurements (λ_max_) in mono-component for CV, MB, RhB, and MG were 596, 661, 554, and 612 nm, respectively. A slight shift in the wavelength can be observed in the multi-dye solution owing to the interaction between the dye molecules.

### 2.3. Adsorption Studies

The present study focused on a comparative analysis of the removal of dyes (individual and simultaneous) using biowastes as adsorbents, i.e., GS, MP, MBARK, and ML. Kinetic, isothermal, and thermodynamic studies were also carried out to understand the mechanism of adsorption by fitting the data into various kinetic, isothermal, and thermodynamic models. For individual adsorption study, the aqueous solutions of CV, MB, RhB, and MG with 10 mg/L concentration were prepared. For a typical study of individual removal of dyes, 10 mg of the adsorbent was weighed and added in 10 mL of dye (adsorbent dosage = 1 mg/mL). The dye solutions were stirred on a rotary shaker for 60 min at 180 rpm. After the completion of 60 min the supernatant of the solution was analyzed using UV–Vis spectrometer to monitor its concentration. All the studies were performed in triplicate sets to ensure the reliability and reproducibility of the findings. From the results obtained from comparative analysis, the best adsorbent (MBARK) was further evaluated for its performance in a multi-component dye system (mixture of dyes) for simultaneous dye adsorption. The adsorption study for mixture of dyes was performed in a similar way as discussed above such that conc. of each dye was 2.5 mg/L (conc. of mixture = 10 mg/L). The % removal (efficiency) and the adsorption at the equilibrium were calculated using the following formula:

$$\%\;\mathrm{adsorption}=\frac{C_{i}-C_{e}}{C_{i}}\times 100$$

where C_i_ = concentration of dye before adsorption;

C_e_ = concentration of dye at equilibrium.

The adsorption at equilibrium will be calculated using:

Q_e_ = V (C_1_ − C_2_) ÷ M;

Q_e_ = adsorption capacity at equilibrium (mg/g);

V = volume of dye solution taken (mL);

M = quantity of adsorbent added (mg);

C_1_ and C_2_ (mg/L) refer to the concentration of dye before and after adsorption, respectively, at the λ_max_.

## 3. Results and Discussion

The biowastes used in this study were characterized by various characterization techniques for obtaining detailed information regarding the material, such as morphology, functional group, particle size, and charge. A detailed discussion of the results obtained is as follows.

### 3.1. Characterization of Adsorbents

**Figure 1:**
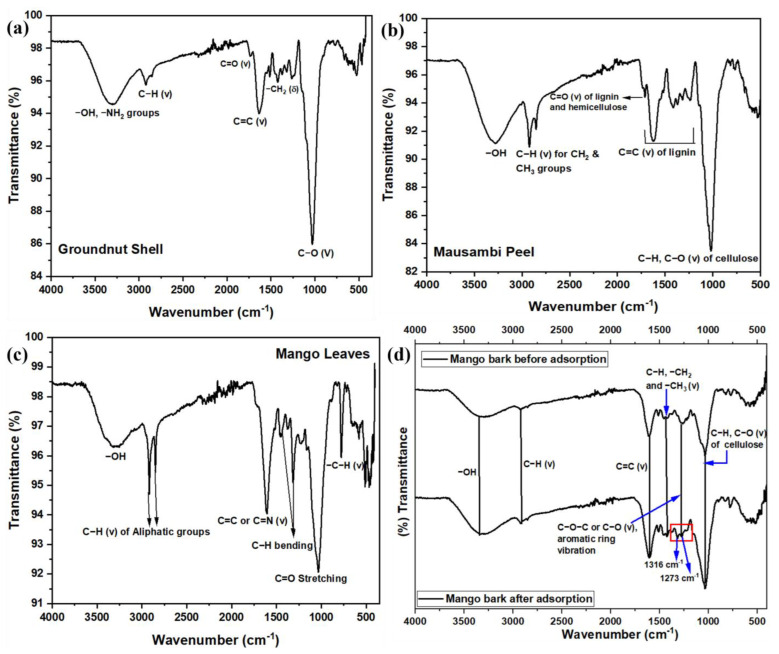
FT-IR spectra for all the adsorbents (**a**) GS, (**b**) MP, (**c**) ML, and (**d**) MBARK before and after adsorption.

Figure 1a–d show the FT-IR spectra for GS, MP, MBARK, and ML. In all of the adsorbents, the distinctive common bands at around 3330 cm^−1^ confirm the presence of the -OH functional group, peaks at around 2923 cm^−1^ indicate the presence of -CH groups, and C=C groups identified at around 1646 cm^−1^ are due to the presence of lignin and cellulose in all adsorbents [43]. The peak around 1023 cm^−1^ represents the presence of C-O bonds for all the adsorbents. The C=O stretch in GS and MP is found at 1738 cm^−1^, whereas in ML, this peak is at 1039 cm^−1^ [25,36]. The -CH_2_ bending in GS is characterized at 1423 cm^−1^. Thus, from the FT-IR spectra, we can confirm that all the biowastes (adsorbents) are rich in lignin, cellulose, and hemicellulose content [32,44].

The best adsorbent for individual removal (MBARK) was further explored for individual dye removal. Since, the major focus of this study is the simultaneous dye removal, the spent MBARK after the adsorption of dyes from the mixture was characterized using FTIR for checking the stability and interaction of dye molecules with adsorbent (Figure 1d. On comparing the peaks of MBARK before adsorption, it was observed that there was a bifurcation in the peak corresponding to the region of C-O-C and C-O stretching into two peaks at 1273 cm^−1^ and 1316 cm^−1^, pointing toward the interaction between oxygen and carbon-containing functional groups with the dye molecules (as marked in Figure 1d) [45,46,47]. The rest of the peaks remained unchanged before and after adsorption, thereby indicating no significant chemical change in the structure of the adsorbent.

**Figure 2:**
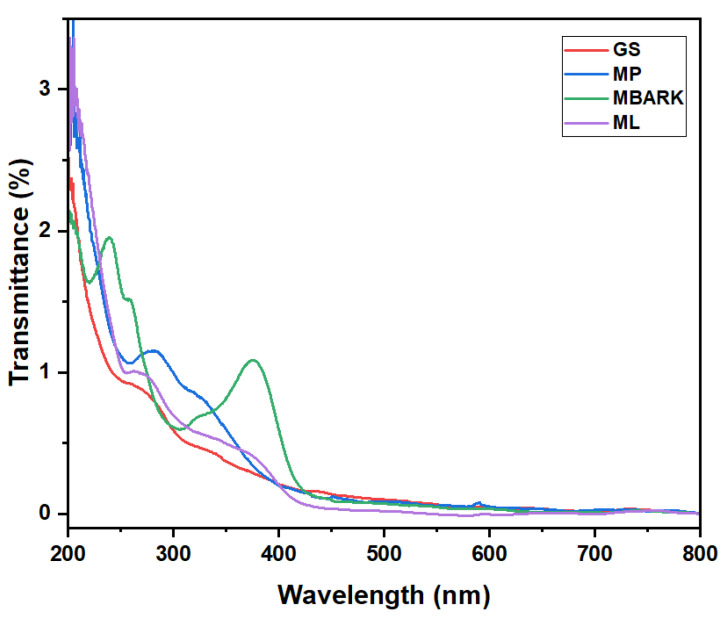
UV–Vis spectra of aqueous solution of Groundnut Shell (GS), Mosambi Peel (MP), Mango Bark (MBARK), and Mango Leaves (ML).

Figure 2 shows the UV–Vis spectra of the adsorbents. The UV spectra of the adsorbents were recorded to check that the adsorbents were not absorbing in the wavelength area corresponding to the CV, MB, RhB, and MG dyes, i.e., in the range from 450 nm to 700 nm. From Figure 2, we can conclude that the adsorbents do not show any peak in the range of the dyes, i.e., from 450 nm to 700 nm, thus causing no interference during the adsorption analysis.

**Figure 3:**
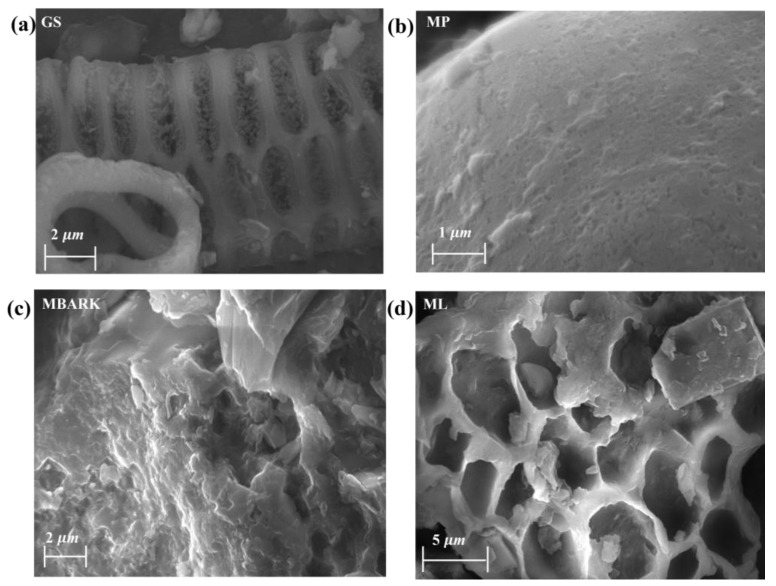
SEM images of the adsorbents (**a**) GS, (**b**) MP, (**c**) MBARK, and (**d**) ML.

Figure 3a–d show the SEM images of GS, MP, MBARK, and ML, respectively. From the images, it can be inferred that most of the adsorbents have porous morphology. The morphology of GS and ML are clearly visible to be porous, whereas MBARK possesses irregular morphology, while MP has a smooth surface with minute pores on it. More images of the adsorbent with different magnifications are provided in Figure S1 (Supplementary Materials).

The particle size of all the adsorbents as analyzed by zeta sizer measurements is shown in Table 2. MBARK has the smallest while GS has the largest particle size. It was estimated that the small particle size of the adsorbent will promote the adsorption process contributing to the removal efficiency.

**Figure 4:**
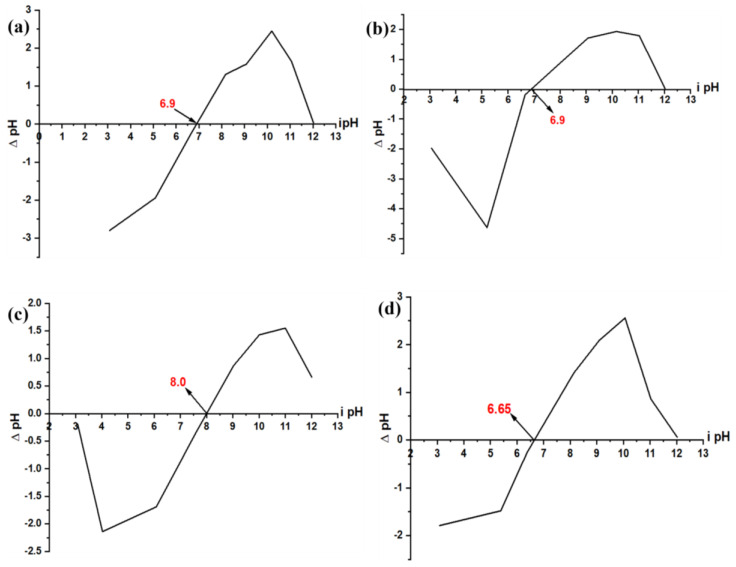
PZC plot for (**a**) GS, (**b**) MP, (**c**) MBARK, and (**d**) ML.

The PZC values for GS, MP, MBARK, and ML were found to be 6.9, 6.9, 8.0, and 6.6, respectively (Table 2). The inference from this PZC analysis is that when the pH values of the aqueous solution are less than the PZC of adsorbents, then the material exhibits a positive charge on its surface, while at pH value more than that of PZC, then the surface has a negative charge. At pH = PZC, no charge is present on the surface of the adsorbent. From the PZC values it was observed that MBARK exhibits the highest PZC compared to all adsorbents. The PZC of any adsorbent is very crucial in determining the adsorption mechanism based on the charge on the adsorbent at a particular pH. It can also be used to predict the optimum pH for a desired analyte to be removed. Figure 4a–d show all the PZC plots for all the adsorbents.

Surface area is considered as the prime aspect for determining the potential of any material as an adsorbent. The results obtained from BET analysis (total surface area, pore size, and pore volume) for all the adsorbents are summarized in Table 2. From the BET analysis, it was observed that the MBARK possesses a high surface area and low pore size as compared to the other adsorbents. This may be due to the difference in composition of woody biomass (MBARK) and non-woody biomass (GS, MP, and ML). For example, certain characteristics and properties of biomass such as the lignin content and bulk density are higher in woody biomass than non-woody biomass. It has also been reported that activated carbon or biochar prepared from woody biomass tends to have a higher surface area as compared to non-woody biomass [48]. It is predicted that the high surface area of MBARK will lead to enhanced removal efficiency.

### 3.2. Adsorption Studies

#### 3.2.1. Individual Dye Removal

**Figure 5:**
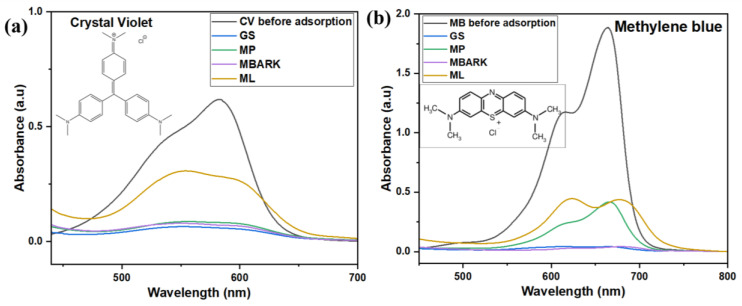

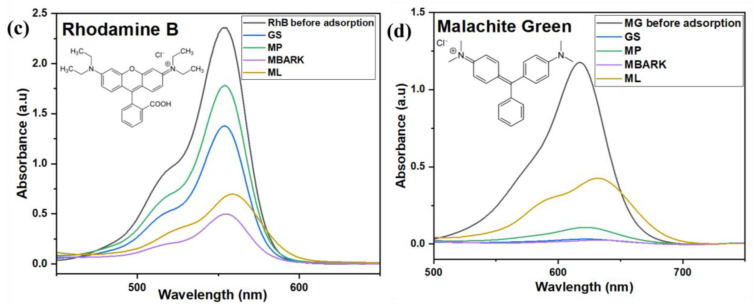
UV–Vis spectra showing adsorption of (**a**) CV, (**b**) MB, (**c**) RhB, and (**d**) MG as a single component system (adsorbent dosage = 1 mg/mL, Dye concentration = 10 mg/L, total contact time = 60 min).

The different biowastes collected were evaluated for the adsorption of various cationic dyes. Figure 5a–d show the UV–Vis spectra of the adsorption of the CV, MB, RhB, and MG. As observed from the spectral results, a drastic reduction in the absorbance values indicates the absorption of dye by the adsorbents. Table 3 represents the % removal of all four dyes by the biowaste-derived adsorbent. From the (%) removal values, it was observed that both GS and MBARK exhibit good overall adsorption efficiency for almost all the dyes. The maximum removal of dyes is exhibited by MBARK. It was found to remove 98.08%, 78.85%, and 98.18% of MB, RhB, and MG, respectively. It was also found that GS exhibits a remarkable removal efficiency of 97.78, 90.39, and 97.3% for the MB, CV, and MG dyes, respectively. Similar mono-component dye removal has been explored using similar biowastes like groundnut shells, neem bark, mango leaf powder, etc. [19,30,37]. The previously reported studies involve either activation of the adsorbent, which adds to the cost, high adsorbent dosage, or more time consumption as compared to the present study. Moreover, dye removal, as a single analyte system only, has only recently been attempted.

##### Kinetic Studies

**Figure 6:**
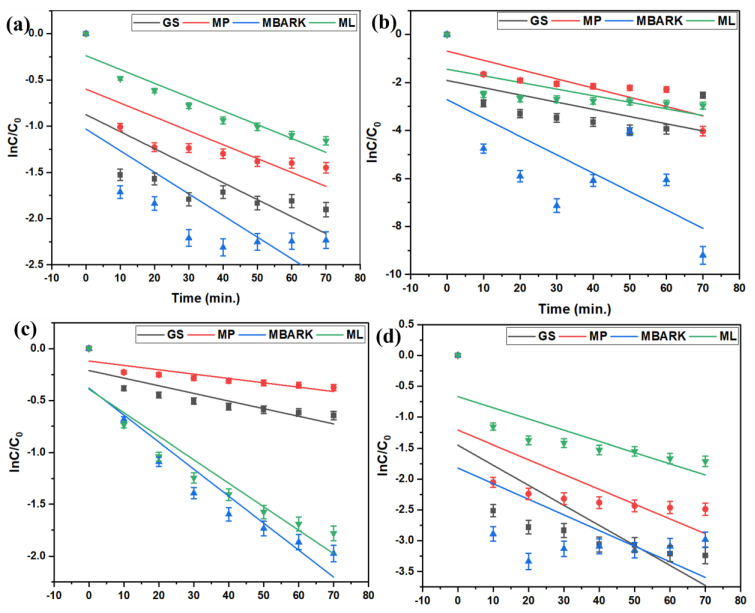
First −order kinetic model for removal of dyes using all four adsorbents: (**a**) CV, (**b**) MB, (**c**) RhB, and (**d**) MG (adsorbent dosage = 1 mg/mL, dye concentration = 10 mg/L, total contact time = 70 min).

**Figure 7:**
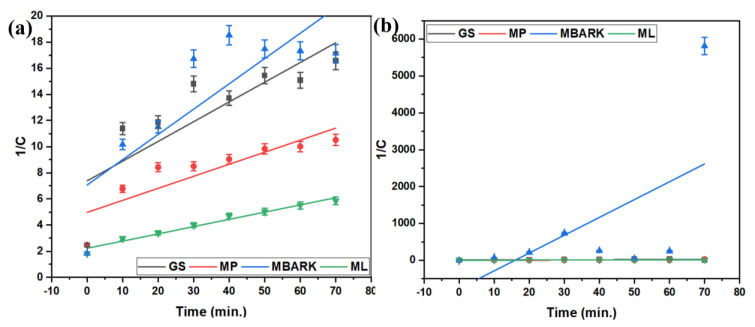

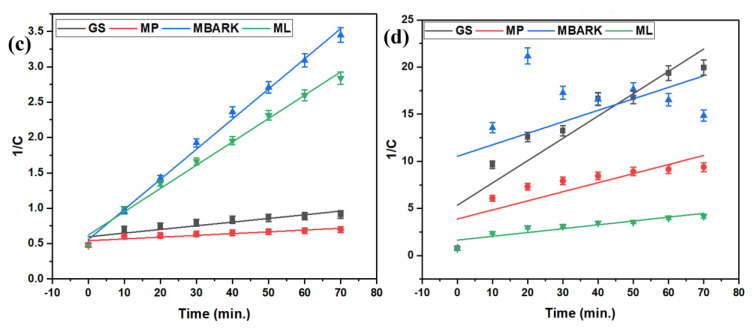
Second−order kinetic model for removal of dyes using all four adsorbents (**a**) CV, (**b**) MB, (**c**) RhB, and (**d**) MG (adsorbent dosage = 1 mg/mL, dye concentration = 10 mg/L, total contact time = 70 min).

**Figure 8:**
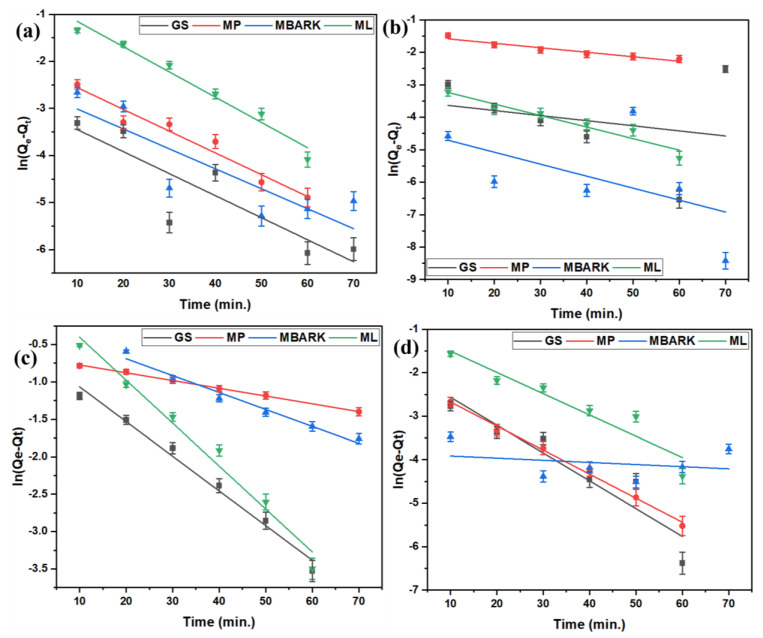
Pseudo −first −order kinetic model for removal of dyes using all four adsorbents: (**a**) CV, (**b**) MB, (**c**) RhB, and (**d**) MG dyes (adsorbent dosage = 1 mg/mL, dye concentration = 10 mg/L, total contact time = 70 min).

**Figure 9:**
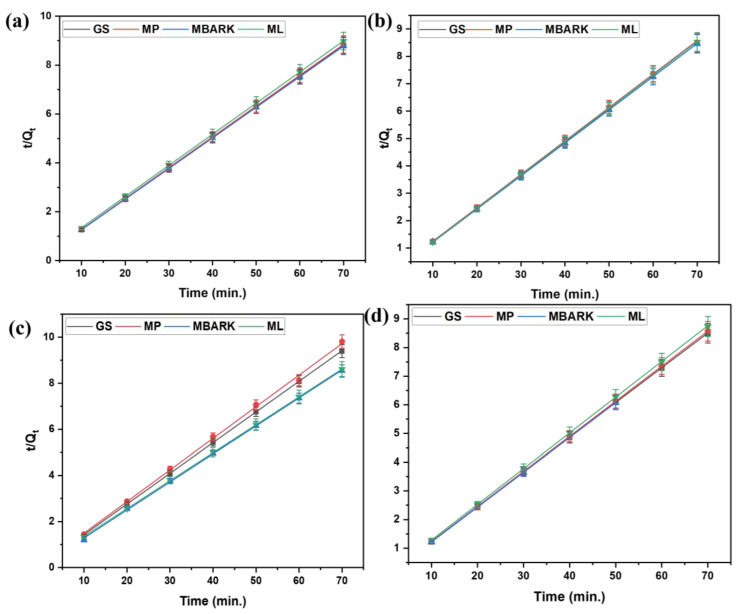
Pseudo −second −order kinetic model for removal of dyes using all five adsorbents: (**a**) CV, (**b**) MB, (**c**) RhB, and (**d**) MG dyes (adsorbent dosage = 1 mg/mL, dye concentration = 10 mg/L, total contact time = 70 min).

For the kinetic studies, the entire process is the same as mentioned in Section 2.3. The supernatant was analyzed at fixed time intervals (10, 20, 30, 40, 50, 60, and 70 min) for monitoring the adsorption equilibrium and nature of dye adsorption. The kinetic data obtained from the study were analyzed by fitting them into four different kinetic models (linear fitting as per equations given in Table 4): First-order (FO), Second-order (SO), Pseudo-first order (PFO), and Pseudo-second order (PSO), as shown in Figure 6, Figure 7, Figure 8 and Figure 9. The parameters, like R^2^, rate constant (k), etc., obtained from the linear fittings of the models, are listed in Table 5. Based on high R^2^ values, it was inferred that the PSO model best fits the present adsorption studies. This hints at the chemisorption process involved in the dye removal [49]. It also states that the adsorption rate of the reaction is dependent on the adsorption capacity of the adsorbent and is independent of the concentration of the analyte (adsorbate).

From the comparative study of all four adsorbents, it was concluded that GS and MBARK showed good overall removal of all the dyes. However, the adsorbent that gave excellent % removal efficiency for all four dyes was MBARK. Thus, MBARK was further explored for its potential as an adsorbent in a more complex matrix. Simultaneous dye removal was carried out using MBARK as an adsorbent. The effects of dosage, pH, and isothermal studies were performed for the same, for a more detailed investigation.

#### 3.2.2. Simultaneous Dye Removal

**Figure 10:**
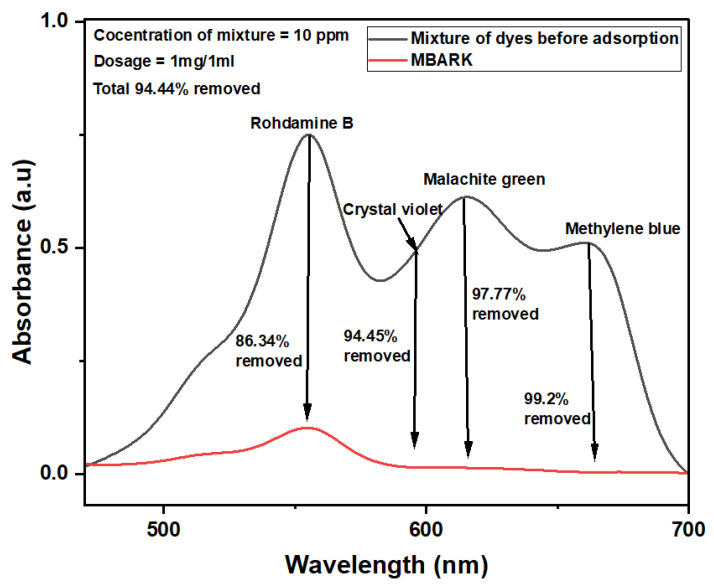
UV–Vis spectra showing removal of CV, MB, RhB, and MG simultaneously from a mixture of dyes using MBARK (adsorbent dosage = 1 mg/mL, dye mixture concentration = 10 mg/L (each dye = 2.5 mg/L), pH of solution = 6, total contact time = 60 min).

As wastewater is a complex matrix containing a mixture of dyes, it is vital to evaluate the adsorption efficiency of adsorbents in a multi-component dye system. The adsorbent derived from Mango bark was used as an adsorbent for the simultaneous removal of dyes from a dye mixture. According to the results of adsorption experiments, MBARK is an excellent adsorbent for removing a mixture of dyes (Figure 10). The removal efficiency of CV, MB, RhB, and MG was almost 94.45% ± 2, 99.02% ± 0.4, 86.34% ± 2, and 97.77 ± 1, respectively. The total overall removal was found to be 94.44% ± 2 in 60 min.

#### 3.2.3. Effect of pH

**Figure 11:**
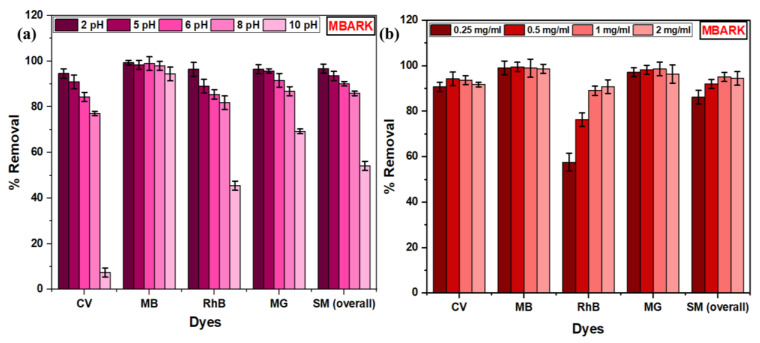
Bar graphs of (**a**) Effect of pH, (**b**) Effect of dosage for MBARK on simultaneous removal of dyes (adsorbent dosage = 0.25 to 2 mg/mL, dye mixture concentration = 10 mg/L (each dye = 2.5 mg/mL), pH = 2 to 10, and total contact time = 60 min).

Adsorption studies were conducted at pH of 2, 4, 7, 8, and 10 to investigate the effect of pH on adsorption efficiency. The pH of a dye solution is an essential factor that affects the adsorption study as it affects the chemistry between adsorbate and adsorbent [50]. The overall % removal by MBARK for the mixture of dyes is 96.69%, 93.52%, 90.08%, 85.88%, and 54.10% for 2, 5, 6, 8, and 10 pH values, respectively. The bar graph for removal of each dye at a different pH is shown in Figure 11a. The highest removal for almost all the dyes was achieved at a pH of 2. At this pH, the adsorbent surface was found to have a positive charge on its surface as inferred from PZC. Thus, this rules out the possibility of adsorption through electrostatic attraction (due to like charges of dye and adsorbent), and the adsorption can probably be due to an ion-exchange mechanism [29]. Another possibility for favored adsorption may be the modification of the adsorbent or dye molecule (due to protonation or deprotonation) at a particular pH, which causes a shift in the pH value of the analyte solution. The UV–visible spectra of individual dyes at a different pH have been taken to monitor the structural changes (Figure S2). The shift in pH after adding the adsorbent (if >PZC) may cause electrostatic interaction between the positively charged dye and negative adsorbent surface. No specific change in % removal for MB, CV, and MG [51,52] was found during the entire pH range, whereas, in RhB, when the pH is increased, the % removal decreases. The reason for this decrease in RhB may be due to the Zwitter ionic structure or electrostatic repulsion between the dye molecule and adsorbent in basic medium [53]. Thus, to summarize, the adsorption process of the studied cationic dyes is mostly governed by ion exchange in acidic pH (when the adsorbent is positively charged) and electrostatic interaction in basic medium (when the adsorbent is negatively charged). Other forces like hydrogen bonding and π–π interaction also participate in the adsorption process, which is discussed in Section 3.2.6.

#### 3.2.4. Effect of Dosage

To determine the ideal dosage for the removal of a mixture of dyes, the effect of adsorbent dosage on the adsorption of dyes was studied. Dosage studies were performed by taking 0.25, 0.5, 1, and 2 mg/mL in a 10 mL dye mixture solution. Figure 11b depicts the bar graph for removal at different dosages of 0.25 mg/mL, 0.5 mg/mL, 1 mg/mL, and 2 mg/mL. MBARK showed an overall removal of 86.08%, 91.98%, 94.98%, and 94.34% for 0.25 mg/mL, 0.5 mg/mL, 1 mg/mL, and 2 mg/mL of adsorbent dosage, respectively. The % removal increased from 86.08% to 94.98% with an increase in adsorbent dosage from 0.25 mg/mL to 1 mg/mL. This can be attributed to the increase in adsorption sites with an increase in dosage. It can also be noted that the removal % remained almost the same when the adsorbent was increased to 2 mg/mL from 1 mg/mL, which indicates agglomeration of the adsorbent particles. Similar results have been reported by Jha et al. [54]. From all the results, the optimal dosage was determined to be 1 mg/mL and was kept the same for all the studies.

#### 3.2.5. Isothermal Studies

For the removal of a mixture of dyes, the isothermal analysis was performed using MBARK as an adsorbent. Isothermal studies were carried out by varying the concentration of the aqueous dye mixture solution. The experimental results were plotted as Q_e_ (adsorption capacity at equilibrium) vs. C_e_ (concentration at equilibrium) as per non-linear fitting equations, as shown in Table 6, to investigate the Langmuir, Freundlich, and Temkin adsorption isotherms.

**Figure 12:**
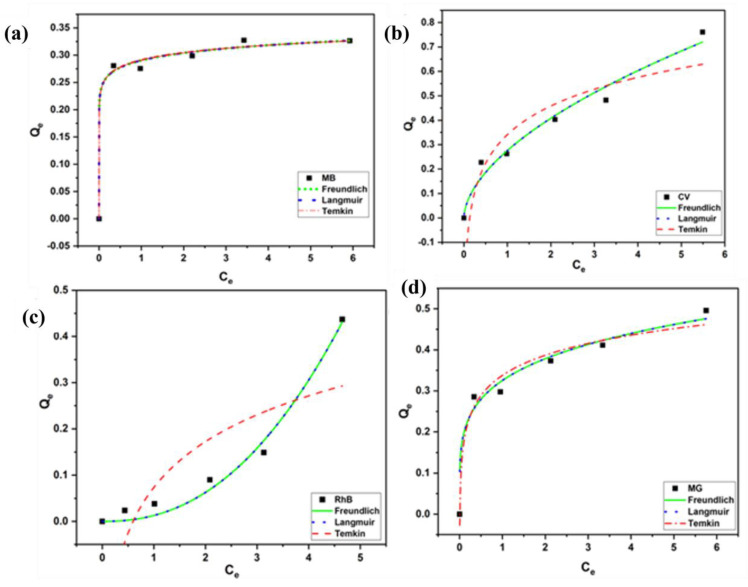
Freundlich, Langmuir, and Temkin isotherm models for (**a**) MB, (**b**) CV, (**c**) RhB, and (**d**) MG dyes using MBARK (adsorbent dosage = 1 mg/mL, dye concentration = 2.5 mg/L to 25 mg/L, contact time = 60 min).

The non-linear fitting was performed for all the isothermal data obtained, as shown in Figure 12. The values of all the isothermal constants derived from the fitting are shown in Table S2 (Supplementary Materials). The results showed that the Langmuir isotherm model provided the closest fit to the data (R^2^ = 0.99) for all the dyes. This suggests that the adsorption occurs at a specific homogeneous site and is limited to one layer only. Further, no more adsorption occurs once this layer is formed. The observations are in agreement with the results obtained in the kinetics study, confirming the possibility of chemisorption. However, for CV, MB, and RhB, the values of R^2^ for Freundlich and Langmuir are close to each other hinting at the chance of the formation of a multi-layer during the adsorption process.

#### 3.2.6. Thermodynamic Study

**Figure 13:**
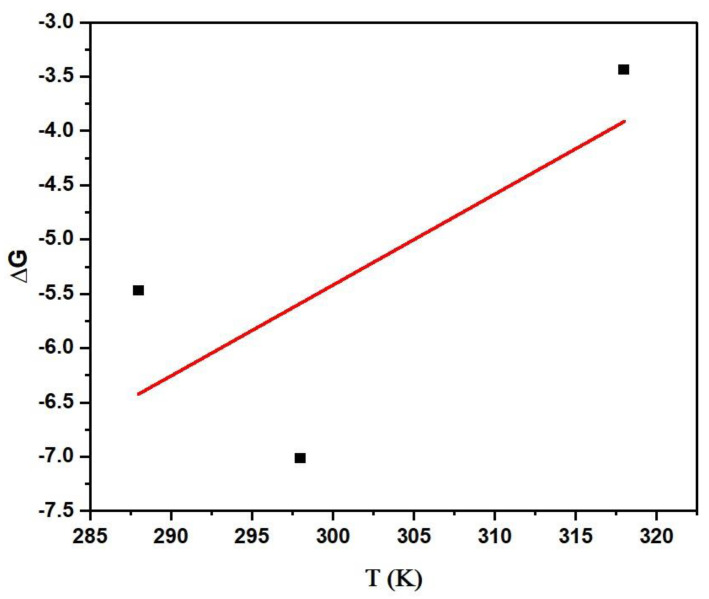
Thermodynamic plot of the simultaneous reaction performed at varying temperatures (adsorbent dosage = 1 mg/mL, dye mixture concentration = 10 mg/L, contact time = 60 min).

The simultaneous removal of dye was performed at three different temperatures (288 K, 298 K, and 318 K) to study the effect of temperature on the performance of MBARK. The data were further plotted to derive the values of enthalpy (ΔH) and entropy (ΔS) from the value of ΔG, as shown in Figure 13. The value of ΔG was calculated using Equation (1). From the calculated ΔG value, a graph of ΔG vs. temperature was plotted according to Equation (2), as shown in the figure.

(1) ΔG=−RTlnk

(2) ΔG=ΔH−TΔS

where R is the gas constant and T is the temperature in Kelvin. The values of ΔG, ΔH, and ΔS calculated from the graph and equation are listed in Table 7. The negative value of ΔG indicates that the reaction is spontaneous at all temperatures, which is supported by the −ve value of ΔG and +ve value of ΔS. The negative value of ΔG indicates that the reaction is exothermic in nature. The value of ΔS is positive, which implies an increase in the randomness during the solid adsorbent −liquid dye phase interaction during the adsorption process [55].

#### 3.2.7. Adsorption Mechanism

**Figure 14:**
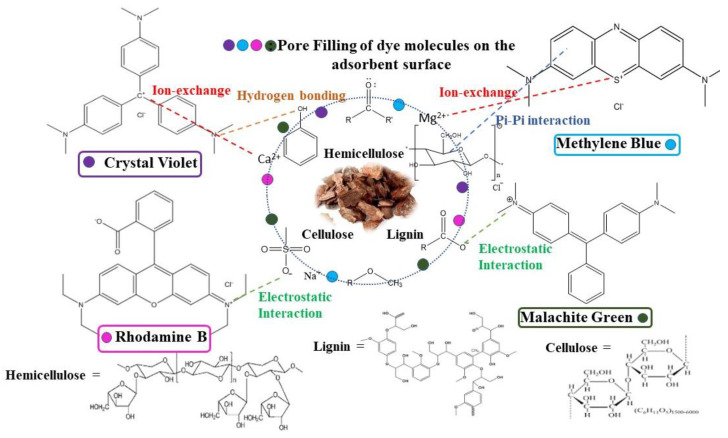
Probable mechanism and interaction based on the functionalities of adsorbent (MBARK) and dye molecule.

It is very crucial to understand the general interactions happening between the analyte and the surface of the selected adsorbent during the adsorption process. Different functional groups present on the adsorbent bind to the molecules of the analyte by interactions, ion exchange, hydrogen bonding, precipitation, and complexation. Various factors like the charge on the dye molecule (cationic/anionic) and adsorbent, aromaticity, and pH determine the dominant mechanism. A schematic representation of the probable interactions occurring between MBARK and the four dye molecules analyzed in this study is shown in Figure 14.

## 4. Regeneration of Adsorbent

The reusability of the adsorbent is the most significant factor to be considered when selecting a suitable candidate for adsorption. Thus, it becomes inevitable to investigate the regenerability of the spent adsorbent from an environmental and economic point of view. In this study, the spent adsorbent (MBARK) was washed with acetone till no dye was desorbed into the acetone. The regenerated adsorbent showed enhanced/comparable removal performance for four cycles. After completion of the fourth cycle, a decrease in removal efficiency (by 5%) was observed. Table 8 shows the % removal of dyes during successive cycles. There was a decrease in overall removal (%) from 94.44% to 92.17% while using the adsorbent for four cycles. The regeneration studies clearly indicate that this adsorbent can be reused up to at least four cycles without much loss in absorption efficiency.

## 5. Conclusions

The present study involves the application of an adsorption technique using four different biowastes as adsorbents (GS, MP, MBARK, and ML) for the removal of dyes as an individual component as well as a multi-component system. A comparative study of all the above-mentioned adsorbents for the removal of CV, MB, RhB, and MG from their aqueous solution was performed. All the adsorbents showed good removal efficiency for all the dyes in 60 min, but maximum removal of 88.42%, 98.08%, 78.85%, and 98.18% for CV, MB, RhB, and MG, respectively, was achieved by MBARK. The process was found to follow the pseudo-second order (R^2^ = 0.99963 to 1), suggesting the involvement of chemisorption in the dye adsorption. MBARK was found to remove all dyes exceptionally well showing a removal of >95% for almost all the dyes. Therefore, it was further evaluated for its performance in simultaneous dye removal from a mixture. MBARK exhibited an overall removal of 94.44% in simultaneous dye removal. Other studies like pH, dosage, isothermal, and thermodynamic studies were also performed for the same. The overall adsorption process was well described by Langmuir isotherm (R^2^ = 0.99), representing monolayer adsorption via chemisorption (from isothermal studies) and the nature of the reaction was found to be exothermic and spontaneous. The adsorbent can be regenerated using acetone and shows effective dye removal for four cycles (92.17%). Thus, this study effectively displayed the suitability and feasibility of the biowastes in treating different dyes, namely, CV, MB, RhB, and MG, proving it to be a sustainable approach to be explored in the future for removing harmful pollutants from wastewater.

## Figures and Tables

**Table 1:** Advantages and disadvantages of various physical and chemical wastewater treatment methods.

Methods	Advantages	Disadvantages	Ref.
Ion exchange	Small amount of sludge	High operation cost	[4]
Coagulation/flocculation	low energy consumption	No complete removal of heavy metals	[5]
Chemical precipitation	Low capital cost	Large amount of sludge	[6]
Flotation	Economically efficient	Low elimination efficiency	[7]
Membrane filtration	High separation selectivity	High operational cost	[8]
Adsorption	Easy and simple operation	Weak selectivity	[9]

**Table 2:** Values of particle size (from Zeta sizer analysis), PZC, surface area, pore size, and pore volume for all adsorbents.

Adsorbents	Particle Size (Z-Average (r.nm))	PZC	BET Surface Area (m^2^/g)	Pore Size (nm)	Pore Volume (cm^3^/g)
GS	748.6	6.9	1.857	12.279	0.009
MP	543.8	6.9	1.106	14.756	0.004
MBARK	533.2	8.0	2.463	11.065	0.007
ML	423.1	6.6	0.986	14.304	0.004

**Table 3:** % Removal of dyes (MB, CV, RhB, and MG) individually for different adsorbents (adsorbent dosage = 1 mg/mL, Dye concentration = 10 mg/L, total contact time = 60 min).

Adsorbents	% Removal			
	MB	CV	RhB	MG
GS	97.78 ± 2	90.39 ± 3	41.49 ± 2	97.3 ± 3
MP	78.01 ± 3	86.57 ± 2	24.34 ± 2	90.93 ± 1
MBARK	98.08 ± 1	88.42 ± 1	78.85 ± 2	98.18 ± 3
ML	78.6 ± 2	54.08 ± 1	71.55 ± 1	67.60 ± 1

**Table 4:** Linear form of equations for different kinetic models.

Kinetic Model	Linear Equation
First order	$\ln C=-kt+\ln C_{0}$
Pseudo-first order	$\ln\left(Q_{e}-Q_{t}\right)=\ln Q_{e}-k_{1}$t
Second order	$\frac{1}{C}=kt+\ln C_{0}$
Pseudo-second order	$\frac{t}{Q_{t}}=\frac{1}{k_{2}Q_{e}^{2}}+\frac{t}{Q_{e}}$

C = concentration of adsorbate after adsorption; k = rate constant; t = time; C_o_ = concentration of the adsorbate before adsorption; Q_e_ = amount of adsorption at equilibrium; Q_t =_ amount of adsorption at any given time.

**Table 5:** The values of k and R^2^ of different kinetic model fittings for the adsorption of different dyes using all the adsorbents (adsorbent dosage = 1 mg/mL, dye concentration = 10 mg/L, total contact time = 70 min).

Dye	Adsorbents	Q_e_(mg/g)	First Order		Second Order		Pseudo—First Order		Pseudo—Second Order	
			k	R^2^	k	R^2^	k	R^2^	k	R^2^
CV	GS	1.62	0.01838	0.51562	0.15087	0.68036	0.04681	0.77812	1.94692	1
	MP	1.53	0.01502	0.60066	0.09197	0.75561	0.04628	0.95367	0.84410	1
	MBARK	1.57	0.02339	0.54096	0.19344	0.68527	0.04239	0.74484	1.34942	1
	ML	0.83	0.01493	0.89678	0.5524	0.975	0.05367	0.96926	0.21479	0.99998
MB	GS	2.4	0.03007	0.32167	0.23314	0.28002	0.01569	0.06546	1.72565	0.99991
	MP	1.66	0.03857	0.74471	0.29097	0.47652	0.01383	0.93041	0.25611	0.99983
	MBARK	2.42	0.07657	0.49181	48.31715	0.35359	0.03688	0.28828	14.47806	1
	ML	1.68	0.02755	0.47033	0.1145	0.70882	0.03557	0.93002	1.54918	1
RhB	GS	0.64	0.00736	0.74293	0.00519	0.82086	0.04637	0.98511	0.18305	0.99996
	MP	0.03	0.0042	0.74749	0.00250	0.79498	0.01038	0.98511	0.14851	0.99859
	MBARK	2.08	0.02602	0.89677	0.04258	0.99531	0.02263	0.97562	0.20824	0.99963
	ML	1.81	0.02264	0.88044	0.03296	0.99111	0.05743	0.97893	0.10184	0.99996
MG	GS	2.68	0.03246	0.54845	0.23663	0.85798	0.06397	0.88389	1.03446	1
	MP	2.46	0.02395	0.48837	0.09636	0.69503	0.0553	0.99402	1.03438	1
	MBARK	2.71	0.02533	0.31694	0.12195	0.2404	0.00489	0.0725	4.30477	1
	ML	1.67	0.0181	0.63893	0.04067	0.83123	0.04891	0.90187	0.30413	0.99998

**Table 6:** Non-linear equations for Freundlich, Langmuir, and Temkin isotherm models.

Isotherm Model	Non-Linear Form
Freundlich isotherm	$Q_{e}=K_{f}C_{e}^{\frac{1}{n}}$
Langmuir isotherm	$Q_{e}=\frac{Q_{max}bC_{e}}{1+bC_{e}}$
Temkin isotherm	$Q_{e}=B\ln\left(K_{T}C_{e}\right)$

Q_e_ = amount of adsorption at equilibrium; C_e_ = concentration of adsorbate at equilibrium; k_f_ = Freundlich adsorption capacity constant; Q_max_ = maximum monolayer adsorption capacity of adsorbent; b = constant related to affinity between adsorbent and adsorbate; B = constant related to heat of adsorption; K_T_ = Temkin isotherm constant.

**Table 7:** Values of different parameters derived from thermodynamic studies for simultaneous adsorption of dyes (adsorbent dosage = 1 mg/mL, dye mixture concentration = 10 mg/L, contact time = 60 min).

ΔG (kJ/mole) at Varying Temperature			ΔH (kJ/mole)	ΔS (J/mole·K)
288 K	298 K	318 K	−30.51	0.0836
−5.47	−7.01	−3.43		

**Table 8:** Removal (%) of different dyes in the mixture up to 4 cycles using spent adsorbent (adsorbent dosage = 1 mg/mL, dye mixture concentration = 10 mg/L, contact time = 60 min).

Dye	Cycle 1	Cycle 2	Cycle 3	Cycle 4
MB	97.24	99.68	97.70	97.71
CV	94.90	99.54	96.39	93.39
MG	86.46	99.45	89.84	82.05
RhB	96.10	90.99	97.01	95.55
Overall	93.67	97.41	95.23	92.17

## Data Availability

The data presented in this study are available on request from the corresponding authors.

## Supplementary Materials

The following supporting information can be downloaded at: https://www.mdpi.com/article/10.3390/toxics12040266/s1, Figure S1. SEM images (a,b) GS, (c,d) MP, (e,f) MBARK, and (g,h) ML; Figure S2. UV-Visible spectra of (a) crystal violet, (b) methylene blue, (c) rhodamine B, and (d) malachite green at different pH; Table S1. Summary of specific adsorbents-removal of dyes, heavy metals and adsorption capacity; Table S2. Value of R2 for the Freundlich, Langmuir, and Temkin isotherm models (adsorbent dosage = 1 mg/mL, dye mixture concentration = 2.5 mg/L to 25 mg/L, contact time = 60 min).
