# Supplementary material for: Sustainable Bio-Based Adsorbents for Simultaneous and Efficient Removal of Hazardous Dyes from Aqueous Solutions
Source: Toxics. 2024 Apr 1;12(4):266. doi: 10.3390/toxics12040266 (PMC11054129; doi:10.3390/toxics12040266)
Supplement: Supplementary file 1 [file toxics-12-00266-s001.zip › toxics-2781213-supplementary.pdf]

Sustainable bio-based adsorbents for simultaneous and efficient removal of hazardous industrial dyes

Dhwani Vara[a,#], Stuti Jha[a,#], Shweta Bisht[b], Syed Shahabuddin [a,*], Rama Gaur [a,*], Suhas[c] and Inderjeet Tyagi[d*],

[a]Department of Chemistry, School of Energy Technology, Pandit Deendayal Energy University, Knowledge Corridor, Raisan, Gandhinagar, 382426, Gujarat, India

[b]Division of Research and Innovation, School of applied and life sciences, Uttaranchal University, Dehradun-248007, Uttarakhand India

[c]Department of Chemistry, Gurukul Kangri (Deemed to be University), Hardwar-249404, Uttrakhand, India

[d]Centre for DNA Taxonomy, Molecular Systematics Division, Zoological Survey of India, M-Block, New Alipore, Kolkata-700053, West Bengal, India

*Correspondence: rama.gaur@sot.pdpu.ac.in(R.G.); syedshahab.hyd@gmail.com; syed.shahabuddin@sot.pdpu.ac.in (S.S.); indertyagi011@gmail.com (I.T.)

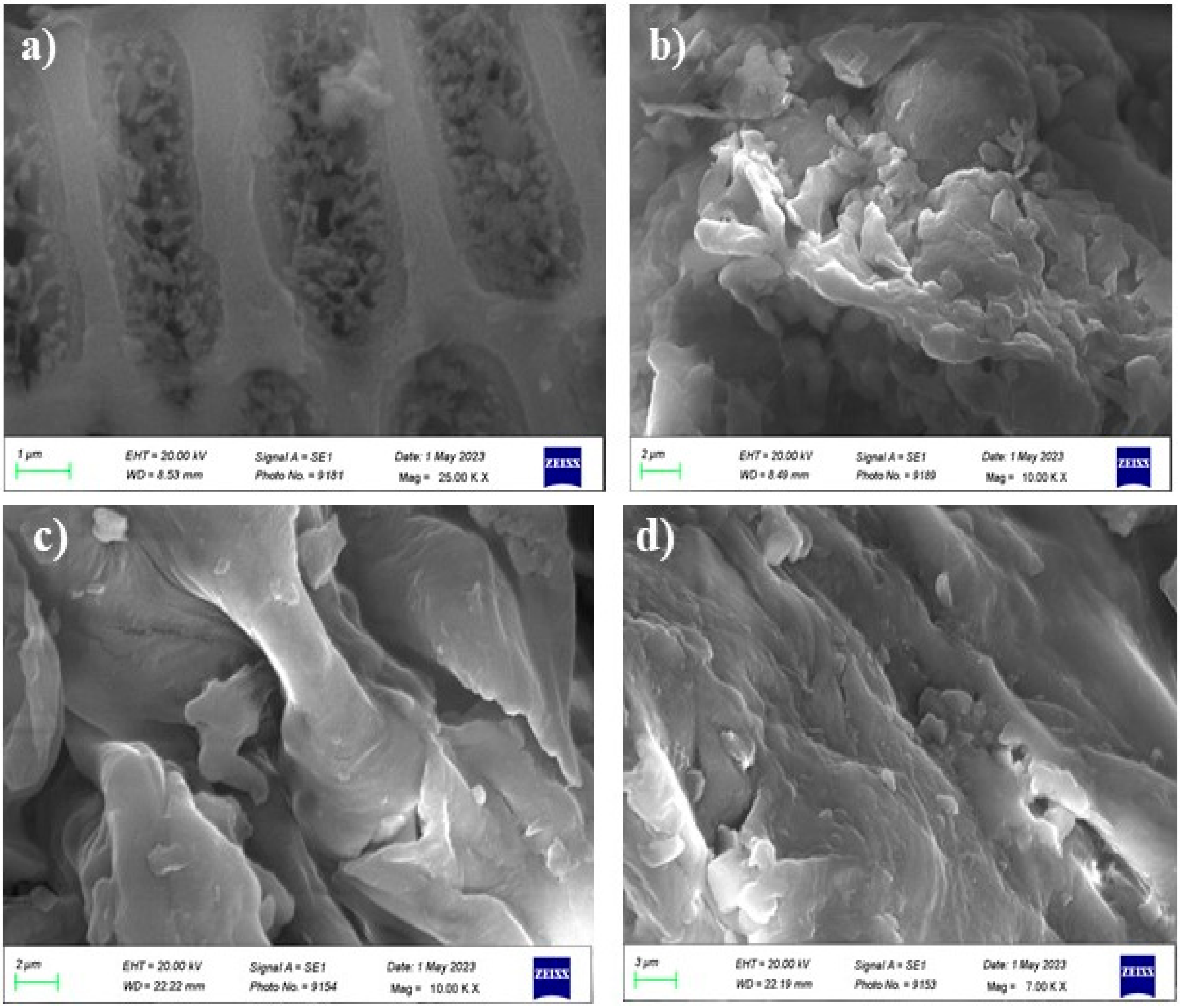

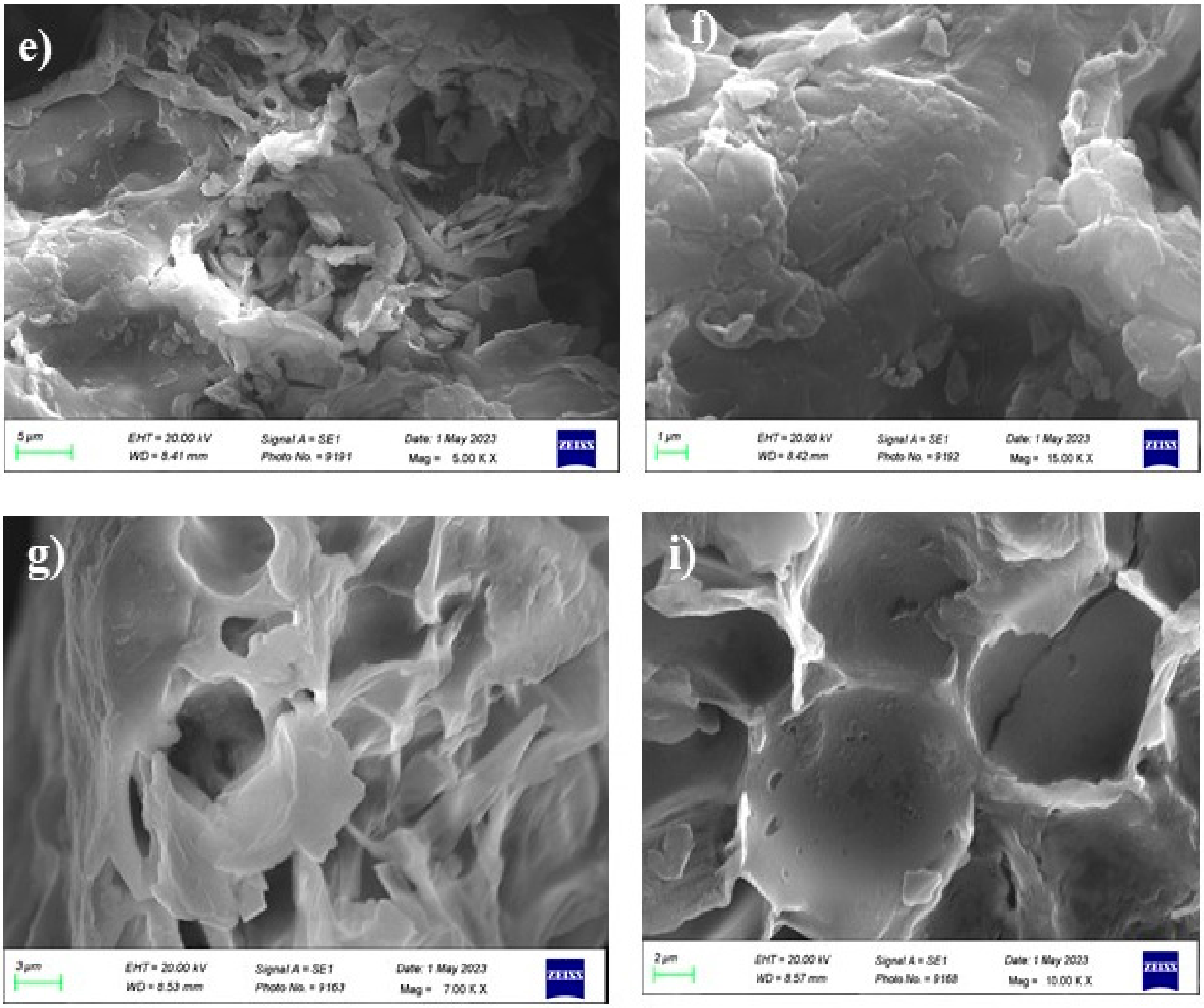

Figure S1. SEM images (a,b) GS, (c,d) MP, (e,f) MBARK, and (g,h) ML.

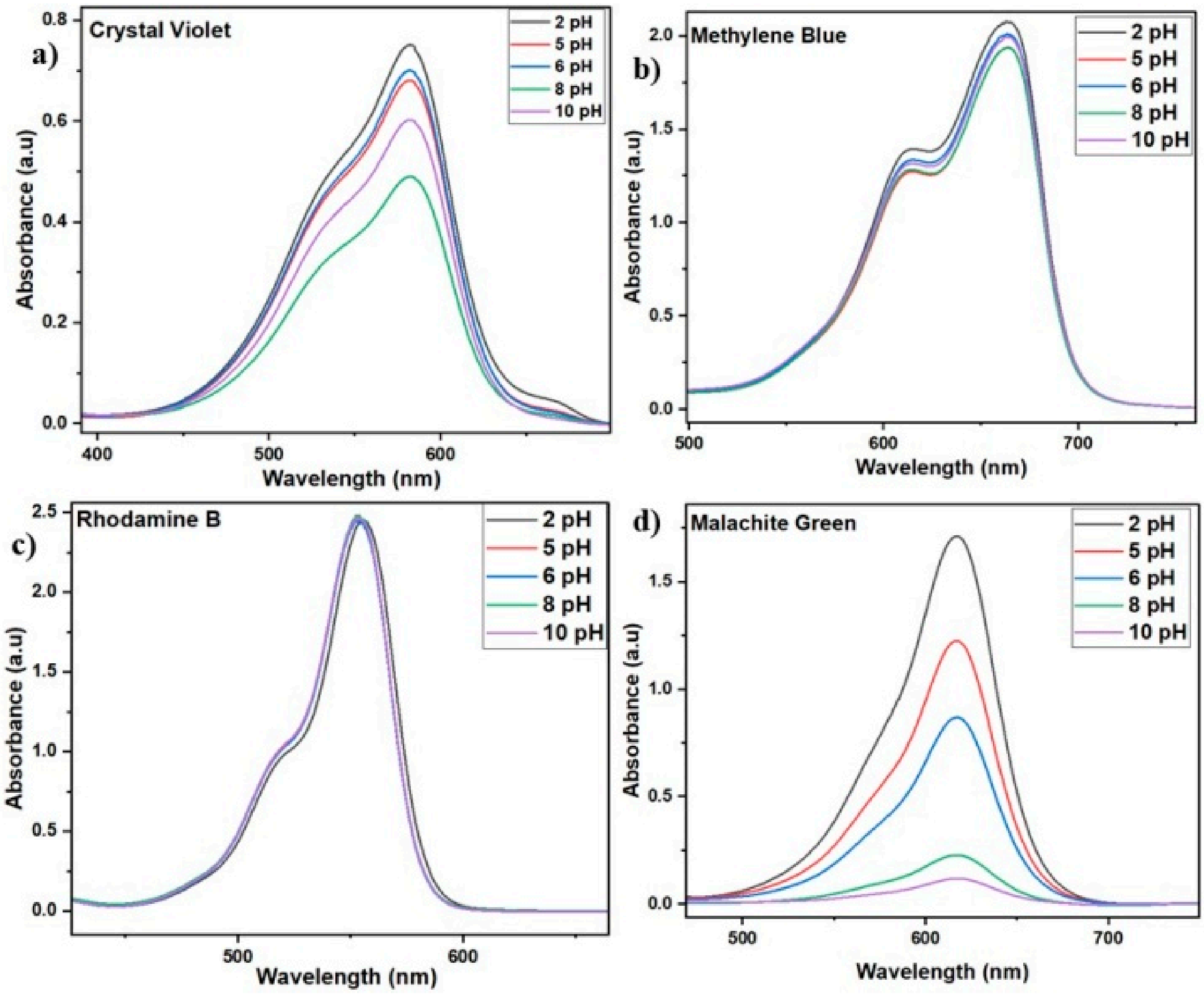

Figure S2. UV-Visible spectra of (a) crystal violet, (b) methylene blue, (c) rhodamine B, and (d) malachite green at different pH.

Table S1. Summary of specific adsorbents-removal of dyes, heavy metals and adsorption capacity.

Sr. No.	Material	Analyte	Dosage mg/ml	Dye Concentration	Contact Time	Adsorption capacity	% Removal	Ref.
**1.**	Groundnut Shells (GNS)	MB	5 mg/L	10 mg/L	24 h	-	99.5 %	[18]
**2.**	Groundnut Shell by $ZnCl_2$ Activation	Fuchsin Acid Dye	0.02 g-0.2 g	100 mg/L	5-120 min.	55.5 mg/g	99 %	[19]
**3.**	Groundnut Shell by H_2SO_4 Activation	Chromium and Nickel	2g	--	60-150 min.	-	-	[20]
**4.**	Groundnut shell-based powdered activated carbon	MG	0.07 g/100 ml	-	30 min.	-	94.5%	[21]
	Commercially available powdered activated carbon		0.07 g/100 ml		15 min.		96%	
**5.**	Peanut shell activated using H_3PO_4	Acid Yellow 36 (AY-36)	2-6 g/L	100 – 250 mg/L	20-150 min.	66.7 mg/g	82%-96%	[22]
**6.**	Activated carbon of mosambi peel (ACMP)	Yellow 2G dye	0.004 g/L	100 mg/L	120 min.	-	96.68 %	[23]
	Stem of cotton (ACCS)						94.12%	
**7.**	Sweet lime (Citrus limetta) peel biocha-450°C	Cr (VI)	0.5 to 5 g/L	20 mg/L	8–24 h	100 mg/g	> 90%	[24]
**8.**	Mosambi Peel dust	Cr (VI)	0.5 g/50 mL	5 mg/L	30 min.	3.623 mg/g	99.58%	[25]
**9.**	Mosambi Peel Activated Carbon	EBT	0.004 g cc-1	50-250 mg/L	120 min.	-	30-93 %	[26]
**10.**	Sweet Lime Peels (SLP)	Cu (II)	0.02 g/mL	100–1000 mg/L	6 h	37.45 mg/g	90 %	[27]
**11.**	Mango Bark Powder	MG	0.5-2 g in 50 ml	-	150 min.	4.22 x 10^3 mol.g^{-1}	~ 70 %	[28]
**12.**	Neem Bark	MG	0.5 gin 50 ml	10^{-4}-10^{-6} mol./L	120 min	0.36 mg g^{-1}	35.5-85.4%	[29]
	Mango Bark				150 min	0.53 mg g^{-1}	40.5-91.5 %	
**13.**	Mango (Mangiferaindica) and	Hg^{2+}	0.1 g	--	4 h	-	73.6-91.5	[30]
		Cr^{3+}					50.2-76.2	
	Neem (Azadirachtaindica) bark	Hg^{2+}					91.4-98.0	
		Cr^{3+}					52.2-73.1	
**14.**	HDTMA-Br-*Mangiferaindica*	Dysprosium (III)	0.05–0.5 g	10–500 mg/L	3 h	55.04 mg/g	65%	[31]
	NaOH-treated bark powder of *Mangiferaindica*						71%	
**15.**	Guava Leaf Biomass	As (III)	9 g	100 mg/L	100 min.	1.05 mg/g	97.2%	[32]
	Mango Bark		9 g			1.35 mg/g	94.4%	
	Bagasse		8 g			1.25 mg/g	95.2%	
**16.**	Mango Leaves Powder	Grey BL Dye	0.1-1.5 g/L	50 and 1000	5 h	-	25.00-63.07%	[33]

				mg/L				
**17.**	Mango Leaf Char	Congo Red Dye	0.5 g/dm,	100 mg/L	-	55.7 mg/g	96.79 %	[34]
**18.**	Mango Leaf Powder (MLP)	Acid Yellow-99 (AY-99)	0.2 g	100 mg/L	160 min.	708.15 mg/g	-	[35]
**19.**	Mango Leaf Powder (MLP)	MB	0.1 to 0.8 g	100 mg/L	2 h	156 mg/g	82-99 %	[36]
**20.**	Mango Leaf Powder (MLP)	RhB	25 g/L	250 mg/L	45 min.	50 mg/L	77%	[37]
**21.**	Coconut-Shell Carbon (CSC)	Pb^{2+}	50 mg	10 to 50 mg/L	3 h	26.50 mg/g	85-96%	[38]
**22.**	Raw Coconut Fiber (Cocosnucifera)	CV	0.05 to 3.5 g	6 to 100 mg/L	10-150 min.	32.8 mg/g	99%	[39]
		CR				27.6 mg/g	82%	
**23.**	Coconut (CocosNucifera l.) Coir Dust	MB	0.05–0.20 g	20 mg/L	20 min.	29.50 mg/g	92.1% to 99.5%	[40]
**24.**	**Ground nut shell, Masombi peel, Mango bark, Mango leave**	**Individual dye removal**	**1mg/mL**	**10 mg/L dye con.**	**60 min.**	**0.8-2.7 mg/g**	**~98%**	**Present study**
	**Mango bark**	**Mixture of MB, MG, CV &RhB dye**					**~95%**	

Table S2. Value of R^2 for the Freundlich, Langmuir, and Temkin isotherm models (adsorbent dosage = 1 mg/mL, dye mixture concentration = 2.5 mg/L to 25 mg/L, contact time = 60 mins).

Dye	Langmuir			Freundlich			Temkin		
	b	Q_{max}	R^2	K_F	n	R^2	B	K_T	R^2
CV	0.009	29.81	**0.99**	0.276	1.775	0.78	0.169	7.509	0.99
MB	0.059	5.134	**0.99**	0.290	15.12	0.94	0.019	4264	0.88
RhB	4.245	303.2	**0.99**	0.012	0.437	0.93	0.142	1.674	0.97
MG	0.029	11.19	**0.98**	0.325	4.593	0.98	0.070	118.0	0.68
